# Erythrocyte, Platelet, Serum Ferritin, and P-Selectin Pathophysiology Implicated in Severe Hypercoagulation and Vascular Complications in COVID-19

**DOI:** 10.3390/ijms21218234

**Published:** 2020-11-03

**Authors:** Chantelle Venter, Johannes Andries Bezuidenhout, Gert Jacobus Laubscher, Petrus Johannes Lourens, Janami Steenkamp, Douglas B. Kell, Etheresia Pretorius

**Affiliations:** 1 Department of Physiological Sciences, Faculty of Science, Stellenbosch University, Private Bag X1 Matieland, Stellenbosch 7602, South Africa; chantellev@sun.ac.za (C.V.); 17795397@sun.ac.za (J.A.B.); 2 Suite 104, 1 Elsie du Toit Street, Mediclinic Stellenbosch, Stellenbosch 7600, South Africa; laubscher911@gmail.com (G.J.L.); wodie@iafrica.com (P.J.L.); 3 PathCare Laboratories, PathCare Business Centre, PathCare Park, Neels Bothma Street, N1 City 7460, South Africa; janami.steenkamp@pathcare.org; 4 Department of Biochemistry and Systems Biology, Institute of Systems, Molecular and Integrative Biology, Faculty of Health and Life Sciences, University of Liverpool, Crown St, Liverpool L69 7ZB, UK; 5 The Novo Nordisk Foundation Centre for Biosustainability, Building 220, Kemitorvet, Technical University of Denmark, 2800 Kongens Lyngby, Denmark

**Keywords:** COVID-19, erythrocytes, platelets, P-selectin, serum ferritin, oxygen saturation

## Abstract

Progressive respiratory failure is seen as a major cause of death in severe acute respiratory syndrome coronavirus 2 (SARS-Cov-2)-induced infection. Relatively little is known about the associated morphologic and molecular changes in the circulation of these patients. In particular, platelet and erythrocyte pathology might result in severe vascular issues, and the manifestations may include thrombotic complications. These thrombotic pathologies may be both extrapulmonary and intrapulmonary and may be central to respiratory failure. Previously, we reported the presence of amyloid microclots in the circulation of patients with coronavirus disease 2019 (COVID-19). Here, we investigate the presence of related circulating biomarkers, including C-reactive protein (CRP), serum ferritin, and P-selectin. These biomarkers are well-known to interact with, and cause pathology to, platelets and erythrocytes. We also study the structure of platelets and erythrocytes using fluorescence microscopy (using the markers PAC-1 and CD62PE) and scanning electron microscopy. Thromboelastography and viscometry were also used to study coagulation parameters and plasma viscosity. We conclude that structural pathologies found in platelets and erythrocytes, together with spontaneously formed amyloid microclots, may be central to vascular changes observed during COVID-19 progression, including thrombotic microangiopathy, diffuse intravascular coagulation, and large-vessel thrombosis, as well as ground-glass opacities in the lungs. Consequently, this clinical snapshot of COVID-19 strongly suggests that it is also a true vascular disease and considering it as such should form an essential part of a clinical treatment regime.

## 1. Introduction

Severe acute respiratory syndrome coronavirus 2 (SARS-Cov-2)-induced infection, leading to coronavirus disease 2019 (COVID-19), is strongly associated with various coagulopathies that may result in thrombosis, thrombocytopenia, or bleeding in the later stages of the disease [1,2,3,4,5,6,7,8,9,10,11,12,13,14]. It is also recognized that vascular changes and thrombotic microangiopathy, diffuse intravascular coagulation, and large-vessel thrombosis are major reasons for a poor prognosis [15]. These comorbidities are linked to multisystem organ failure, as well as pulmonary vascular endothelialitis [15]. The presence of endotheliopathy, in particular, is likely to be associated with critical illness and death [16]. In addition, in COVID-19, peripheral lung ground-glass opacities are an accompaniment in mild, moderate, and severe diseases, as visible on computed tomographic (CT) images. Peripheral lung ground-glass opacities in COVID-19 also meet the Berlin criteria for acute respiratory distress syndrome (ARDS) [15]. In a recent study by Pretorius and colleagues, microclots were also present in platelet-poor plasma (to which thrombin was not added) of COVID-19 patients, where they form “spontaneously” [17]. Fluorescent microscopy analysis, with the addition of thioflavin T (ThT) (an amyloid protein marker), confirmed that these microclots were in fact amyloid in nature [17]. Previously, we have found that amyloid fibrin(ogen) is a feature of a variety of inflammatory diseases [18,19,20,21,22,23].

Recently, we discussed the apparent paradox in COVID-19 where both clotting and bleeding can be observed as part of the pathology [14] and proposed that the resolution of the paradox is that these clotting and bleeding phases are separated in time. The later bleeding propensity is mediated by the earlier clotting-induced depletion of both fibrinogen and of von Willebrand factor (VWF). Additionally, central to COVID-19 pathology is the dysregulation of P-selectin [24]. P-selectin is an inflammatory coagulation biomarker involved in clotting, and it is known to modulate interactions between blood cells and endothelial cells [25]. This biomarker (also known as CD62P) can either be inside the platelets or endothelial cells, on platelet membranes, or in its soluble form (sP-selectin) as a circulating plasma biomarker. P-selectin is constitutively present in alpha (α)-granules inside platelets, also in Weibel-Palade bodies inside endothelial cells, or as a platelet membrane receptor after the release from the platelet granules [25,26,27]. As membrane receptor, P-selectin acts as an adhesion receptor to support leukocyte rolling and emigration at sites of inflammation [27]. Soluble P-selectin is alternatively spliced, lacks the transmembrane domain, and plays an important role in modulating interactions between blood cells and endothelial cells [25]. Thus, P-selectin may play a crucial role in COVID-19 endotheliopathy, as well as platelet hyperactivation [16]. In COVID-10, P-selectin may significantly contribute to the adhesion of both pathological and possibly also healthy erythrocytes to damaged endothelia, as well as to adjacent erythrocytes and, also, to hyperactivated platelets. It is known that P-selectin may mediate such an interaction in sickle cell disease.

Recently, Manne and coworkers found distinct changes in the gene expression profiles of circulating platelets of COVID-19 patients [28]. They also found that platelets from COVID-19 patients aggregated faster and showed increased spreading on both fibrinogen and collagen. In addition, the authors suggested that an increase in platelet activation and aggregation could partially be attributed to increased mitogen-activated protein kinase (MAPK) pathway activation and thromboxane generation [28]. Strongly associated with the COVID-19 coagulopathies is the presence of hyperferritinemia. Iron and serum ferritin (a marker of oxidative stress) [29,30] in circulation can act as a marker of damaged cells [31]. Excess circulating iron has long been known to cause blood to clot into an anomalous form [30], later shown to be amyloid in nature [18,19,20,21,22,23,32]. Both platelets and erythrocytes also show pathology in the presence of increased iron and serum ferritin [33,34]. Hyperferritinemia is also known to accompany ultrastructural changes in both platelets and erythrocytes [33,34,35,36,37,38].

P-selectin, serum ferritin, and pathological changes in platelets and erythrocytes may be central to the development of thrombotic pathophysiology and contribute to the decrease in oxygen saturation levels commonly seen during COVID-19. Here, we propose that there is a causality between increased concentrations of serum ferritin and P-selectin, platelet hyperactivation, and their interactions with erythrocytes. In our recent review [14], we conclude that structural pathologies found in platelets in particular, together with spontaneously formed microclots, may be central to vascular changes and thrombotic microangiopathy, diffuse intravascular coagulation, and large-vessel thrombosis, as well as ground-glass opacities in the lungs. Consequently, this clinical snapshot of COVID-19 strongly suggest that it is also a true vascular disease and treating it as such should form an essential part of a clinical treatment regime.

## 2. Results

### 2.1. Blood Proteins Results

Control and COVID-19 patient laboratory results are shown in Table 1. Newly diagnosed COVID-19 patient blood samples were collected, and the blood analysis reported here was done on treatment-naïve patients. The parameters were all done on admission (thus, symptomatic patients at different grades of severity) and at several points during admission. In our experience, hypercoagulability (as shown with thromboelastography (TEG^®^) and PFA200) could certainly predict the patients with the highest risk of developing the severe disease. Of the 37 patients, 15 were classified as having the mild disease and 22 as moderate-to-severe symptoms (based on CT scans); the latter group of patients were in the ICU. Please see raw data link for the spreadsheet of the patient data.

We age-matched COVID-19 (mean age: 53.1 y) and healthy individuals (mean age: 55.6 y) (*p* = 0.6; data normally distributed and analysis done using unpaired *t*-test). Oxygen saturation was low in COVID-19 patients 94.6% (91–96%). Oxygen saturation of healthy individuals are usually between 97% and 100%. COVID-19 patients also have significantly raised C-reactive protein (CRP) and serum ferritin levels (see Table 1).

### 2.2. Fluorescent Microscopy Results

**Figure 1:**
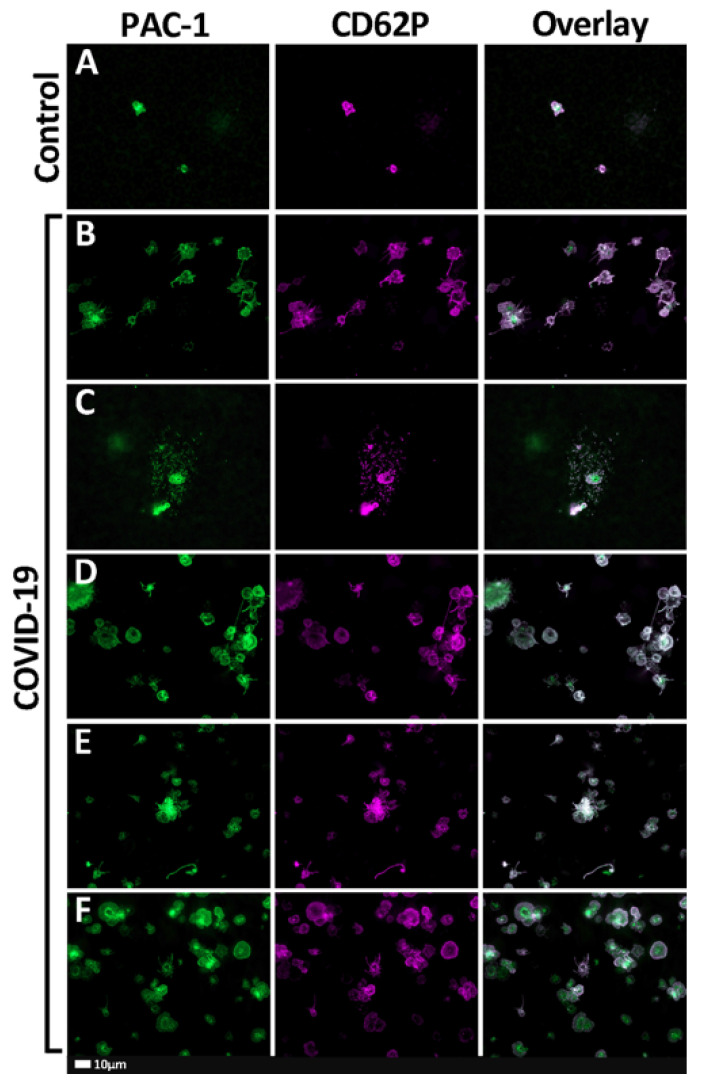
(**A**–**F**) Representative fluorescent micrographs of whole-blood samples stained with PAC-1 (green fluorescence) and CD62P-PE (purple fluorescence). The last column represents an overlay of the two micrographs. (**A**) Representative healthy (control) platelets. (**B**–**F**) Representative fluorescent micrographs from coronavirus disease 2019 (COVID-19) patients.

Figure 1A–F show the representative fluorescent microscope micrographs of healthy and COVID-19 samples stained with PAC-1 (green fluorescence—activated GPIIb/IIIa) and CD62P-PE (purple fluorescence—platelet surface P-selectin). Control platelets are typically small, round cellular entities with only a few pseudopodia extending from their surfaces. Platelets from COVID-19 patients showed extensive platelet hyperactivation; in some instances, severe aggregation and spreading were seen (Figure 1B–F).

### 2.3. Scanning Electron Microscopy Results

**Figure 2:**
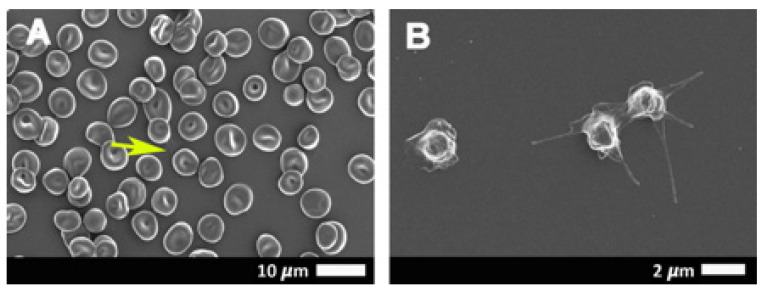
Representative scanning electron micrographs of the erythrocytes (**A**) and platelet ultrastructure (**B**) seen in healthy individuals. Yellow arrow points to a platelet in a whole-blood smear.

**Figure 3:**
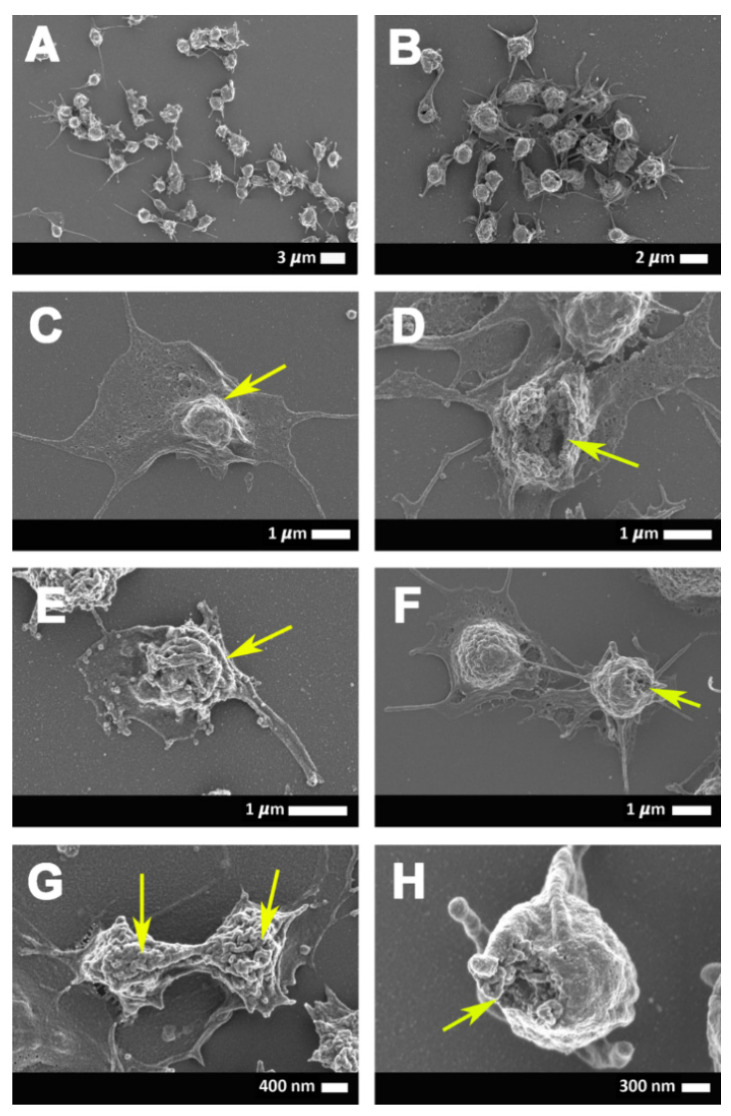
(**A**–**H**) Representative scanning electron micrographs of the platelet ultrastructure seen in COVID-19-positive patients. Yellow arrows show some platelet membranes damage that was observed.

Figure 2A,B shows scanning electron micrographs from healthy erythrocytes (Figure 2A) and platelets (Figure 2B), while Figure 3A–H show platelets from COVID-19 patients. Results from SEM analysis support the fluorescence microscopy results seen in Figure 1, where severe platelet pathologies (spreading and clumping) were visualized with PAC-1 and CD62P-PE. Extensive hyperactivation and clumping were noted in the platelet ultrastructure (Figure 3A,B), with platelet membrane damage, where the membranes are fragmented and granular, were also seen (Figure 3C–H: yellow arrows). We propose that the significant platelet pathophysiology seen in both Figure 1 and Figure 3 suggest that the sP-selectin, which we would expect to be in circulation, may either be bound to receptors on platelets (and other blood cells, including possibly also endothelial cells) or P-selectin may still be present on the platelet membrane where it acts as a binding receptor, facilitating binding between adjacent platelets, red blood cells (RBCs), and also, endothelial cells. This explains the significantly lower sP-selectin values that we report here in Table 1 and confirms the results from Goshua and coworkers in 2020 [16].

**Figure 4:**
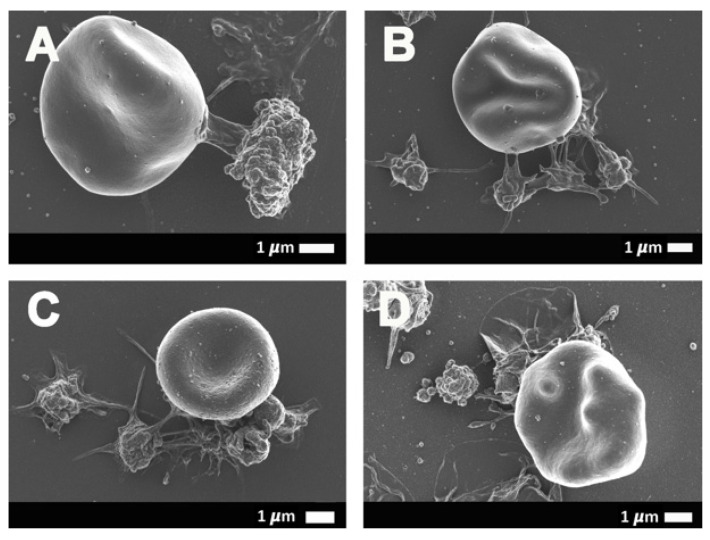
(**A**–**D**) Representative scanning electron micrographs of the interaction between the erythrocytes and platelets of COVID-19-positive patients.

**Figure 5:**
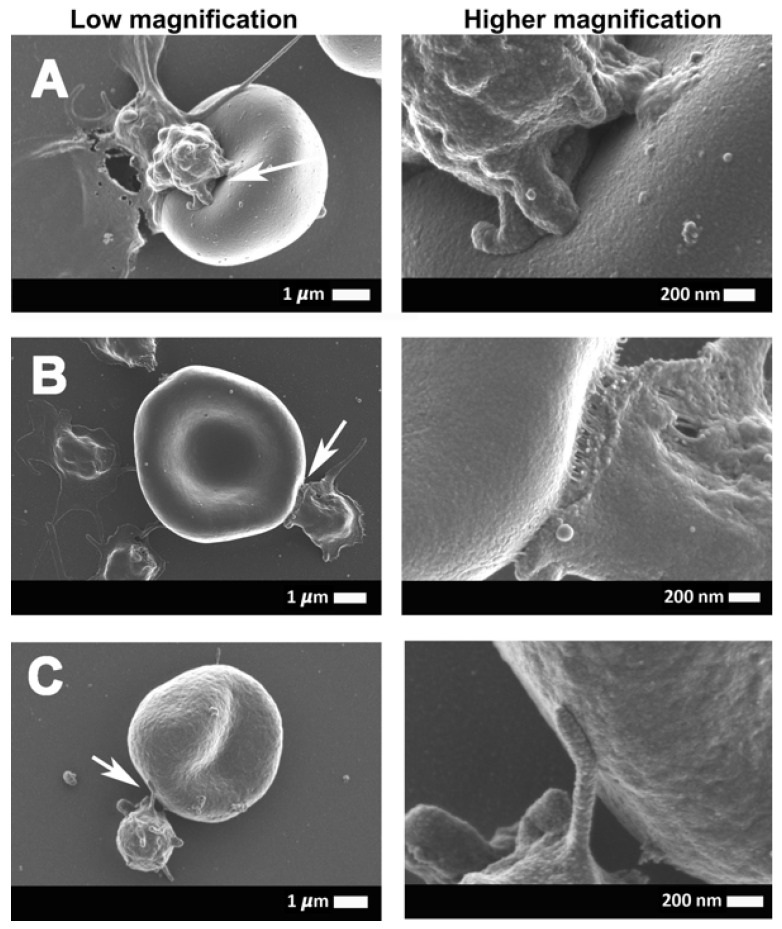
(**A**–**C**) A continuation of the representative scanning electron micrographs of the interaction between the erythrocytes and platelets of COVID-19-positive patients. (**A**–**C**) (column on the left) shows the low magnification micrographs of the platelet-erythrocyte interactions, with the corresponding high magnification micrographs (column on the right) to show the ultra-structures of the interactions. Arrows point to the area that was focused on.

Figure 4 and Figure 5 show the erythrocyte and platelet interactions in COVID-19-positive whole-blood (WB) samples. Figure 5A,C,E shows low magnification micrographs of the platelet-erythrocyte interactions, while the corresponding micrographs in the right column (Figure 5B,D,F) are higher magnification micrographs of the same erythrocyte and platelet to show the ultrastructure of the interactions. In control WB smears, healthy erythrocytes have a typical discoid shape, and platelets are small, round cellular elements that will show slight pseudopodia formations due to contact activation in the glass coverslip on which the WB samples are placed, as seen in Figure 2A,B. In WB samples from COVID-19 patients, minimal erythrocytes show slight eryptotic changes, where the cells do not have the typical discoid shape (Figure 4A,B,D). Hyperactivated platelet and erythrocyte interactions frequently occur in WB smears, where both the erythrocyte and the platelet membranes are fused with each other. Such fusions between membranes can be facilitated by P-selectin when present on the platelet membrane. When on the platelet membrane, P-selectin is known to act as adhesion receptor, where it promotes platelet-platelet, platelet-endothelial cells, or platelet-erythrocyte adhesions [14,39].

**Figure 6:**
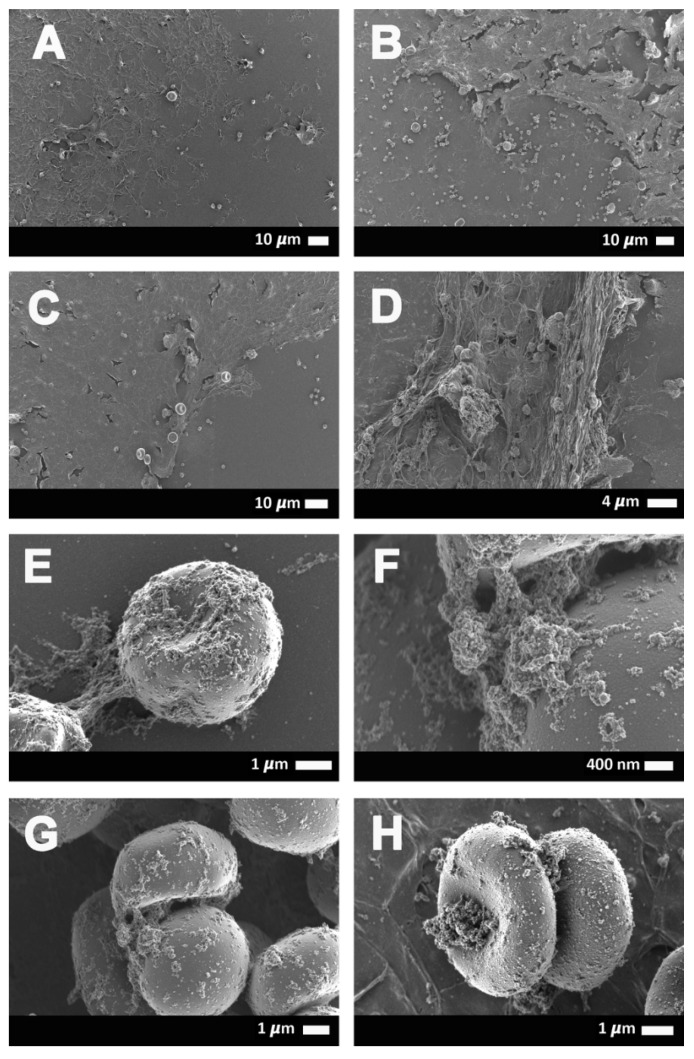
(**A**–**D**) Representative scanning electron micrographs of COVID-19-positive samples with spontaneous fiber-like clotlet formations in close proximity to erythrocytes in whole-blood smears, as well as occasional granular deposits (**E**–**H**) on the erythrocytes.

Figure 6A–H shows erythrocyte ultrastructure and fibrin(ogen) deposits in COVID-19 samples. Figure 6A,D indicates representative WB smears from COVID-19 patients that show the extent of the spontaneously formed fibrin(ogen) deposits. Figure 6E–H are micrographs to show how, in some instances, the fibrin(ogen) deposits aggregate to erythrocytes. We previously reported that spontaneous clotlets are present in platelet-poor plasma (PPP) of COVID-19 patients and that these clotlets have an amyloid structure, as shown using the fluorescent stain ThT [14]. Here, we show these deposits with SEM micrographs, where we find these amyloid deposits in a WB smear (without the addition of thrombin). In healthy individuals, only erythrocytes and platelets will be present in such a smear. However, in COVID-19 patients, clotlets are visible as both fiber-like and, in some instances, granular deposits (see Figure 6).

### 2.4. Viscoelasticity and Viscometry Results

Viscoelasticity and viscometry were also done on PPP samples to determine if the fibrin(ogen) deposits noted in the scanning electron micrographs may indeed affect the clotting parameters, as well as the viscosity of the blood. These results are shown in Table 1. Plasma from COVID-19 patients were significantly more viscous than that of healthy individuals (*p* = 0.02) when measured with the viscometer. Similarly, the TEG^®^ results indicated that the plasma from COVID-19 patients were significantly hypercoagulable compared to healthy plasma. Three TEG^®^ parameters that were significantly increased confirmed the clotting pathology. The parameters include the maximal amplitude (MA), which reflects the ultimate strength of the fibrin clot, as well as the overall stability of the clot. The larger the MA, the more hypercoagulable/stronger the clot. The total thrombus generation (TTG) parameter, indicative of clot strength, and the amount of total resistance (to movements of the cup and pin) generated during clot formation were also significantly increased. In addition, the time parameter, the maximum rate of thrombus generation (MRTG), was also significantly decreased in COVID-19 plasma. MRTG is an indication of the maximum rate of the clot growth. These parameters point to a hypercoagulable clot pathology in COVID-19 patients and confirm our previous results, as well as underscore the ultrastructural changes noted in WB smears [17].

## 3. Discussion

In this current COVID-19 pandemic, the race for answers of how this virus interacts with the body is one of the crucial research questions that need to be answered to obtain a clearer image of how the virus works, as well as make it possible to find treatment regimens that can assist in a COVID-19 patient’s survival. In our current research, it comes as no surprise that serum ferritin levels are increased in COVID-19 blood samples, as it is known as a marker of damaged cells [31]. Its presence in the circulation may cause pathology to platelet erythrocytes, as well as the plasma protein fibrinogen [18,19,20,21,22,23,32,33,34,35,36,37,38]. Iron can also leak into the circulation due to cellular damage to not only erythrocytes but, also, endothelial and other cells. Both iron and serum ferritin may result in the Fenton reaction that may drive oxidative stress in circulation [29,31,40]. Central to COVID-19 pathology is a decreased oxygen saturation, and this was also noted in our COVID-19 sample, and oxidative stress may be a fundamental driver of this clinical parameter. The increase in the CRP levels further confirms the increased inflammatory conditions (hypercoagulability and activated platelets) in COVID-19 patients, as CRP is an acute marker of inflammation but, also, plays a role in the acute-phase immune response in the body [41].

An interesting observation was that the COVID-19 individuals have a decreased P-selectin concentration compared to our control sample (see Table 1). Our COVID-19 concentrations are broadly comparable to those found by [16] (our median concentration was 17.4 ng·mL^−1^ vs. results from two COVID-19 patient groups of the Goshua et al. paper, which were 15.9 ng·mL^−1^ and 11.2 ng·mL^−1^, respectively). However, our sP-selectin concentrations in controls were much higher: 26.7 ng·mL^−1^ and, rather, compared well to that of the results of the ELISA assay kit insert (Goshua et al. paper) that reported a control mean of detectable concentrations (ng·mL^−1^) of 25.8 ng·mL^−1^ for citrate plasma analysis [42]. Our ELISA assay kit control ranges are considered to be 15 ng·mL^−1^ to 55 ng·mL^−1^ (personal communication FineTest Technical Department). Our sample of COVID-19 patients showed extensive platelet pathophysiology, as seen with fluorescent microscopy, as well as SEM analysis. P-selectin may be present in various parts of the blood fraction, either inside platelets and endothelial cells, as a binding receptor on platelet membranes, or as sP-selectin in circulation, where it may act as a biomarker that can activate receptors on either platelets or other cells (including the endothelial cells). We report extensive P-selectin signals on platelets from COVID-19 patients and, therefore, suggest that one reason might be because P-selectin is still present as an adhesion receptor attached to the platelet membrane, where it assists in platelet aggregation. Another reason may be that P-selectin was shed as sP-selectin but that it is now bound to receptors on activated platelets (and other cells where it has receptors, e.g., endothelial cells). This will result in a decrease of sP-selectin but explains the significant signal present on large platelet aggregates and, also, on microparticles, which are visible both with the SEM, as well as the fluorescent microscope. Manne and coworkers suggested that COVID-19 is associated with platelet hyper-reactivity, which may contribute to COVID-19 pathophysiology [28], and our results support their findings. SEM also revealed a noteworthy presence of spontaneous fiber-like clotlet deposits in the WB smears, with the occasional granular deposits on the erythrocyte membranes. Previously, we reported that platelet-poor plasma of COVID-19 patients had a significantly increased amount of amyloid clotlets [17]. Our TEG^®^ and viscometry results also showed that our COVID-19 samples were significantly hypercoagulable and more viscous than that of healthy PPP. The various plasma proteins and their effects on blood clotting and viscosity, together with the presence of the noteworthy activated and aggregated platelets—that is, the cause of platelets and erythrocytes to form aggregates in WB may be the direct cause of vascular pathologies noted in the lungs of COVID-19 patients [14,17].

## 4. Materials and Methods

### 4.1. Ethical Statement

Prior to sample collection, consent was obtained from all participants. The Health Research Ethics Committee (HREC) of Stellenbosch University approved the study (reference number: 9521). This laboratory study was carried out in strict adherence to the International Declaration of Helsinki, South African Guidelines for Good Clinical Practice and the South African Medical Research Council (SAMRC), Ethical Guidelines for Research.

### 4.2. Patient Samples

#### 4.2.1. Covid-19 Patients

Thirty-seven COVID-19-positive samples (18 males and 19 females) were obtained, and blood samples collected before treatment was embarked upon. Sample analyses that were included in this paper were on treatment-naïve COVID-19 patients. Whole-blood (WB) samples were collected from newly diagnosed patients before treatment, and platelet-poor plasma (PPP) were also prepared by centrifuging samples at 3000× *g* for 15 min. Platelet-poor plasma was stored at −80 °C until necessary for analysis (ELISA, thromboelastography, and viscometry); then, the PPP was allowed to thaw until it reached room temperature. The WB samples were prepared on the same day for fluorescent and scanning electron microscopy analyses.

#### 4.2.2. Healthy Samples

Our healthy PPP samples were 13 age-matched controls (5 males and 8 females) previously collected and stored in our plasma repository. They were nonsmokers, with C-reactive protein (CRP) levels within normal ranges, and none were on any anti-inflammatory medication.

### 4.3. Fluorescence Microscopy

Two fluorescent antibodies, CD62P (platelet surface P-selectin) and PAC-1 (activated GP IIb/IIIa), were added to WB to study platelet activation [43]. CD62P is found on the membrane of platelets and the move to the surface of the platelet membrane. This translocation happens after platelet P-selectin is released from the cellular granules during platelet activation. The antibody PAC-1 detects the neoepitope of active GPIIb/IIIa. PAC-1 antibody binding is correlated with platelet activation.

We added 4 µL of PAC-1 (FITC-conjugated) (340507, BD Biosciences, San Jose, CA, USA) and 4 µL CD62P (PE-conjugated) (IM1759U, Beckman Coulter, Brea, CA, USA) to 20-µL WB and incubated the samples for 30 min (protected from light) at room temperature. A 10-µL drop of blood was then placed on a microscope slide, and a coverslip was then placed on the drop of blood. Following preparation, the samples were viewed using a Zeiss Axio Observer 7 fluorescent microscope with a Plan-Apochromat 63x/1.4 Oil DIC M27 objective (Carl Zeiss Microscopy, Munich, Germany). For the PAC-1 marker, the excitation wavelength was set at 450 to 488 nm and the emission at 499 to 529 nm, while the excitation for the CD62P was 540 to 570 nm and the emission 577 to 607 nm. Unstained samples were also prepared with COVID-19 WB to assess any interference from autofluorescence.

### 4.4. Scanning Electron Microscopy

Whole-blood samples were also prepared for scanning electron microscopy (SEM) analysis to assess ultrastructural changes of the erythrocytes and platelets. Ten-microliter WB was placed on a 10-mm round glass coverslip and exposed to 0.075-M sodium potassium phosphate buffer (PBS). Standard SEM preparation steps were followed, including fixing in 4% formaldehyde and a secondary fixation of the sample in 1% osmium tetroxide (OsO_4_). Dehydration steps in an increasing series of ethanol was followed, with the final step, hexamethyldisilazane (HMDS). The samples were coated with carbon and viewed with a Zeiss MERLIN field emission-SEM with the InLens detector using 1 kV (Carl Zeiss Microscopy, Munich, Germany).

### 4.5. P-Selectin ELISA, CRP, and Serum Ferritin

We analyzed soluble P-selectin concentrations in PPP samples of 10 control and 30 COVID-19 samples using the EH3818 Human sSELP (soluble P-Selectin) ELISA Kit (FineTest, Wuhan, China). Serum ferritin and CRP concentrations were measured at the PathCare pathology laboratory in Stellenbosch, Western Cape, South Africa. CRP was measured on the AU480 Beckman Coulter machine (Brea, CA, USA) and serum ferritin on the DXI Beckman Coulter machine (Brea, CA, USA). CRP data was cross-checked using a VPLEX multiplex assay (Vascular Injury Panel (V-plex) 2 (human) kits, catalogue number: K15198D, Meso Scale Discover (MSD), Rockville, MD, USA).

### 4.6. Thromboelastography (TEG^®^)

TEG^®^ was performed to assess the clot kinetics and viscoelastic properties of PPP samples from COVID-19 patients (*n* = 7) and healthy control subjects (*n* = 10). Samples were prepared by the addition of 20-μL 0.2-M calcium chloride (CaCl_2_) (7003, Haemonetics^®^, Niles, IL, USA) to a disposable TEG^®^ cup (HAEM 6211, Haemonetics^®^, Niles, IL, USA), followed by the addition of 340 μL of citrated PPP. CaCl^2^ was added to reverse the anticoagulant action of sodium citrate (recalcification of blood) and, consequently, activate the coagulation cascade. Samples were loaded in the measuring channels of the TEG^®^ 5000 Hemostasis Analyzer System (07-033, Haemonetics^®^, Niles, IL, USA), and analyses were performed at 37 °C. TEG^®^ parameters measured included the reaction time (R, minutes), alpha-angle (α, degrees), maximum amplitude (MA, mm), maximum rate of thrombus generation (MRTG, dynes/cm^2^/s), time to maximum rate of thrombus generation (TMRTG, minutes), and total thrombus generation (TTG, dynes/cm^2^) (see Table 2 for the various parameters of this method).

### 4.7. Viscometry

Plasma viscosity was measured with the RheoSense microVISC™ (RheoSense Inc., San Ramon, CA, USA) viscometer, which uses viscometer/rheometer On-a-Chip (VROC^®^) microfluidic sensor technology. Prior to analysis, stored PPP aliquots from SARS-CoV-2 patients (*n* = 7) and healthy control subjects (*n* = 10) were thawed form −80 °C to room temperature (~19 °C). Between each measurement, the microviscometer was cleaned with 1% Scienceware^®^ Aquet^®^ liquid detergent solution (Z273260, Sigma-Aldrich, St. Louis, Missouri, USA) in order to maintain stable viscosity measurements. Plasma viscosity was calculated according to Newton’s law of viscosity:

μ = τ/(γapp)(1)

where μ = viscosity, τ = shear stress, and γapp = apparent shear rate.

### 4.8. Statistics

Statistical analysis was done using GraphPad Prism 8 (version 8.4.3, San Diego, CA, USA). The Shapiro-Wilks normality test was done on all data. Parametric datasets were analyzed using an unpaired *t*-test, and the data expressed as mean ± standard deviation. Nonparametric data sets were analyzed using the Mann-Whitney U test, and the unpaired nonparametric data (all two-tailed) were expressed as medians (Q1–Q3). Raw data of the results can be viewed at the following link: https://1drv.ms/u/s!AgoCOmY3bkKHisVGQ63lwdCsZBlimA?e=4lWhlw.

## 5. Conclusions

Although progressive respiratory failure is seen as a primary cause of death in COVID-19 patients, respiratory failure may be significantly linked to microclots in the circulation and, specifically, in the lungs. Relevant molecular players, particularly P-selectin, may be important early markers of severe vascular disease risk, particularly if soluble P-selectin levels are decreased, as we noted in our results. In addition, the observation of structural changes in the plasma, where microclots and large platelet aggregates are visible, may also lead to important point-of-care analyses that may assist clinicians to plan clinical interventions relating to anticoagulation therapies. Although a high-end fluorescence microscope was used in this research, less expensive fluorescence microscopes may be used as a quick-point point-of-care method. Consequently, fluorescence microscopy, together with thromboelastrography and viscometry, might provide clinicians with critical information in real time about the functioning of platelets and plasma-clotting proteins. COVID-19 is, therefore, a true vascular disease, and treating it as such should form an essential part of a clinical therapeutic regime.

## Figures and Tables

**Table 1:** Sample demographics and biomarker levels (bold indicates significance).

Demographics			
*p*-value (data normally distributed; unpaired *t*-test)		0.6	
Mean age of healthy individuals (*n* = 13)		55.6 (±10.7)	
Mean age of COVID-19 (*n* = 37)		53.1 (±14.7)	
**Serum Ferritin (µg·mL^−1^) (COVID-19: *n* = 33; controls: *n* = 13)**			
*p*-value (Mann-Whitney U test, unpaired nonparametric data expressed as median) (Q1–Q3)		0.0009 (***)	
Median of healthy individuals		113.5 (55.8–149.3)	
Median of COVID-19 patients		306.6 (162.0–699.0)	
**CRP (µg·mL^−1^) (COVID-19: *n* = 37; controls: *n* = 13)**			
*p*-value (Mann-Whitney U test, unpaired nonparametric data expressed as median) (Q1–Q3)		<0.0001 (***)	
Median of healthy individuals		0.8 (0.3–2.1)	
Median of COVID-19 patients		44.1 (13.2–108.0)	
**Soluble P-selectin (ng·mL^−1^) (COVID-19: *n* = 30; controls: *n* = 10)**			
**Our P-selectin (ng·mL^−1^) analysis**		**Goshua et al., 2020 analysis (ng·mL^−1^)**	
*p*-value	0.0009 (***)	*p*-value	0.001
Median of healthy individuals (*n* = 10)	26.7 (23.9–27.2)	Median of healthy individuals (*n* = 13)	9.5 (8.5–11.3)
Median of COVID-19 patients (*n* = 30)	17.36 (14.3–22.3)	Median of ICU COVID-19 patients (*n* = 48)	15.9 (4.8)
		Median of non-ICU COVID-19 patients (*n* = 20)	11.2 (3.1)
Our ELISA kit average ranges for control data	15–55 (Citrate and EDTA plasma)	Their ELISA kit insert average for control data	25.8 (citrate plasma) and 18.3 to 57.4 (EDTA plasma)
**Platelet-poor plasma viscosity (mPa·second)**			
*p*-value (data normally distributed; unpaired *t*-test) (control: *n* = 10; COVID-19: *n* = 7)		0.007 (**)	
Mean viscosity of healthy individuals		2.0 (±0.2)	
Mean viscosity of COVID-19 samples		2.8 (±0.8)	
**Thromboelastography^®^ of platelet-poor plasma (PPP)**			
**Parameter**	**Plasma from healthy samples (*n* = 10)**	**Plasma from COVID-19 samples (*n* = 7)**	***p*-value**
**R**	16.41 (±6.1)	12.9 (±5.2)	0.1
**α angle**	52.4 (34.4–61.3)	46.0 (33.9–63.3)	0.7
**MA**	32.1 (24.5–36.0)	48.2 (35.0–53.8)	0.04 (*)
**MRTG**	4.7 (±3.0)	7.5 (±3)	0.04 (*)
**TMRTG**	17.6 (13.1–23.6)	13.2 (11.3–18.4)	0.4
**TTG**	237 (162.6–415.6)	577.7 (422.0–642.3)	0.009 (**)

Statistical significance was established at *p* < 0.05. (* = *p* < 0.05, ** = *p* < 0.01, and *** = *p* < 0.001). Data is represented as either mean ± standard deviation or median (Q1–Q3). CRP: C-reactive protein, COVID-19: coronavirus disease 2019, R: reaction time, α: alpha, MA: maximal amplitude, MRTG: maximum rate of thrombus generation, TMRTG: time to maximum rate of thrombus generation, and TTG: total thrombus generation.

**Table 2:** Definitions of the various thromboelastography (TEG^®^) parameters, adapted from [23].

TEG^®^ Parameters	Explanation
R value: reaction time measured in minutes	Time of latency from start of test to initial fibrin formation (amplitude of 2 mm); i.e., initiation time.
Alpha-angle (α, degrees)	The rate of fibrin crosslinking indicated by degrees.
MA: maximal amplitude measured in mm	Maximum clot size: it reflects the ultimate strength of the fibrin clot, i.e., overall stability of the clot. The larger the MA, the more hypercoagulable the clot.
Maximum rate of thrombus generation (MRTG) measured in Dyn·cm^−2^·s^−1^	The maximum velocity of clot growth observed or maximum rate of thrombus generation using G, where G is the elastic modulus strength of the thrombus in dynes per cm^−2^.
Time to maximum rate of thrombus generation (TMRTG) measured in minutes	The time interval observed before the maximum speed of the clot growth.
Total thrombus generation (TTG) measured in Dyn·cm^−2^	The clot strength: the amount of total resistance (to movement of the cup and pin) generated during clot formation. This is the total area under the velocity curve during clot growth, representing the amount of clot strength generated during clot growth.

## Abbreviations

COVID-19	Coronavirus disease 2019
sP-selectin	Soluble P-selectin
SEM	Scanning electron microscope
TEG^®^	Thromboelastography
R value	reaction time measured in minutes
Alpha-angle	(α, degrees)
MA	Maximal amplitude measured in mm
MRTG	Maximum rate of thrombus generation, measured in Dyn·cm^−2^·s^−1^
TMRTG	Time to maximum rate of thrombus, measured in minutes generation
TTG	Total thrombus generation, measured in Dyn·cm^−2^
